# An Unforeseen Glimmer of Hope

**DOI:** 10.4269/ajtmh.20-0891

**Published:** 2020-12

**Authors:** Madhusudan Samprathi

**Affiliations:** Rainbow Children’s Hospital, Bangalore, India

“Our brightest blazes of gladness are commonly kindled by unexpected sparks.”

—Samuel Johnson, *The Idler*

One such spark that awaited us was the primary health center (PHC) in a small town called Misrod in Madhya Pradesh, a state in central India. Behind this eye-opening encounter was a collaboration between the Pediatric Intensive Care Unit at the Postgraduate Institute of Medical Education and Research, Chandigarh; UNICEF; and the National Health Mission (a public health initiative by the government of India to improve health care in underserved areas). This project aims to train pediatricians working in the peripheral health centers of the state in managing acutely ill children. This state, called the “heart of India” owing to its geographical location, unfortunately, grapples with one of the country’s highest neonatal and infant mortality rates.

The abbreviation “PHC” in several low- and middle-income countries usually brings to one’s imagination an image of a dilapidated building, ill-kempt environs, stray dogs loitering on the corridors, rusted cots, and malfunctioning equipment. But the PHC at Misrod seemed to be an exception, right from the word go. A closer look at the bright name board at the gate showed that it is the first ISO-9001–recognized PHC in the region. ISO-9001 is an internationally recognized standard for quality management systems issued by the International Organization for Standardization.

The medical officer, the man behind the transformation this PHC has witnessed in the last 4 years, welcomed us with folded hands and a warm smile. As he led us inside, one could not help notice the modest yet exceptionally clean corridors and rooms. Bright posters imparting powerful, crisp messages on the importance of hygiene graced the spaces above windows. “Those spaces actually looked very ugly before we covered them with these posters,” he laughed. Many such posters carrying messages related to maternal and childcare were also seen on the walls, at a much lower level than one would expect. “Posters on walls are hardly read by patients, as they spend more time sitting on the benches waiting for their turn, we thought posters stuck lower down at the eye level are more likely to be read!” he smiled.

One corner of the corridor served as a small makeshift emergency room with a bed (that could be folded if needed for a child), an examination lamp (that was strategically attached to one limb of the crash cart), and the Emergency Triage and Treatment chart on the wall. Another tidy room with five beds served as the ward. The adjoining room was a kitchen where food was cooked for the patients and also doubled up as the nurses’ duty room.

Along the stairs to the first floor, the wall was adorned with sprucely set frames with visitors’ feedback. With encouraging words from officers of neighboring villages to experts from world-renowned universities like Johns Hopkins, the wall literally sung praises to the healthcare team!

The first floor of the building housed a laboratory which performed basic tests. One corner of the floor, earlier a dump yard for waste, had been renovated to a tidy seminar room. Other than training peripheral workers about aspects of health care, the medical officer, along with two other volunteers, helped them become more literate. “Early marriages and financial troubles forced several of them to discontinue formal education but could not dampen their desire to learn. We try to fulfill their long unrealized wish,” he remarked.

As we strolled around the PHC, he recalled his initial days. “Before I took over 4 years back, the area surrounding the PHC was a junkyard; we used to spend the first few hours every day cleaning the place, picking up broken bottles, and other litter. Snakes were a common sight; I myself have killed three!” Small glass bottles originally bought with laboratory reagents in them, now hung prettily with ornamental plants in the garden. The space behind the building was now a kitchen garden brimming with plants of several varieties. At the corner was a fish tank with *Gambusia* fish which devour mosquito larvae, another example of adept use of available space.

Being a government institution, how did they manage the funds? “Part of it, we manage to get, sometimes after repeated requests. But for several smaller things, we pool in money and manage ourselves!” he explained. “Managing and motivating the staff is a huge challenge, I must admit. Patients directly giving their positive feedback to my staff has made a huge difference; my team members feel elated! Knowing that your work has directly made a difference to somebody’s life is probably the best incentive!” he beamed.

As we sat down on the cozy sofa in his room, the homely ambience was hard to miss. As though reading our minds, he remarked, “For me it is important that all my team members feel ‘at home’; we spend a significant part of our lives at our workplace, so why not make it an enjoyable experience?” Directly above his chair, where government offices typically hang portraits of politicians, was framed the Indian tricolor flag in all its splendor. Every inch of space had been used to the maximum, and every element resonated with the joy of giving.

En route to the airport after the 5-day trip, we marveled at the city’s largest lake, whose waters were shimmering in the first rays of the morning sun. Every center and every individual we had met had reflected the undying human desire to do better. A desire continuously nurtured by grit, patience, and dedication that remained uncowed in the face of adversity. The new, exciting possibilities that had unfolded delighted our minds. At the same time, the overwhelming fear of the huge task of training pediatricians working in the peripheral health centers challenged the intellect. But the one surreal fragrance that lingered on was the sheer creativity and dynamism we had seen at Misrod, which soothed our souls!

